# The ethnic profile of patients with birthmarks reveals interaction of germline and postzygotic genetics

**DOI:** 10.1111/bjd.15260

**Published:** 2017-04-21

**Authors:** S. Polubothu, V.A. Kinsler

**Affiliations:** ^1^ Genetics and Genomic MedicineUCL Institute of Child HealthLondonU.K; ^2^ Paediatric DermatologyGreat Ormond St Hospital for ChildrenLondonU.K

dear editor,* GNA11* and *GNAQ* are highly homologous genes encoding different Gα subunits of heterotrimeric G‐proteins. We recently described postzygotic activating mutations in *GNA11* or *GNAQ* as causes of phakomatosis pigmentovascularis (PPV), and *GNAQ* mosaicism as a cause of extensive dermal melanocytosis (EDM).^1^ *GNAQ* mosaicism has previously been found to cause Sturge–Weber syndrome (SWS) and isolated nonsyndromic port‐wine stain‐type capillary malformations.^2^ In all of these clinical phenotypes the mutations almost exclusively affect codons 183 of the protein products Gα_q_ and Gα_11_.

What is not yet understood is how the same mutations can lead to such differing skin phenotypes, either vascular alone (SWS), pigmentary alone (EDM) or a combination of both (PPV). The existence of a common precursor cell leading to both the vascular and pigmentary birthmarks is likely, as the same mutation has been identified in both types of lesion in patients with PPV. In mosaic disorders we would usually therefore invoke the issue of the timing of the mutation as the cause of differing phenotypes, or in other words that the mutation leading to PPV would be expected to occur earlier in embryogenesis than that for SWS or EDM. If this were the case we would expect the multiorgan phenotype in PPV to be more severe than in SWS, owing to an earlier embryological mutation. This was suggested in one case series of PPV;^3^ however, owing to the well‐known phenomenon of dermal melanocytosis being overlooked as a normal finding by examining doctors,^4^ it is likely that PPV is currently underdiagnosed in comparison with SWS, and publications relating to PPV may therefore be biased towards severe cases. Even if timing of the mutation proves to be important once larger cohorts are collected, germline genetic factors could also contribute to the differences in phenotype observed from the same mutations.

Looking at our patient cohorts we hypothesized that ethnicity may be associated with a phenotype‐modifying effect in this spectrum of diseases. Although ethnicity is a loosely defined classification, it is already known to be clearly associated with self‐resolving dermal melanocytosis (Mongolian blue spots), which is far more common in Afro‐Caribbean than white populations.^4^ A bias towards nonwhite ethnicity was suggested in one previous study of PPV,^3^ but it was not systematically studied against control populations.

Ethnicity data are collected routinely upon attendance at our hospital, where patients and families choose their own ethnicity from a standard list. We have assumed that there should be no inherent bias in this choice relating to the type of birthmark with which the child presents. Ethnicity data were extracted for all patients seen in our department over a 2‐year period, between March 2011 and March 2013, with a diagnosis of SWS, isolated facial port‐wine stain, congenital melanocytic naevi, congenital epidermal naevi and infantile haemangioma. For the rarer conditions PPV and EDM, where patient numbers were small, we obtained information relating to all patients with the condition rather than restricting our observations to this time period, to maximize the size of this cohort.

**Figure 1:**
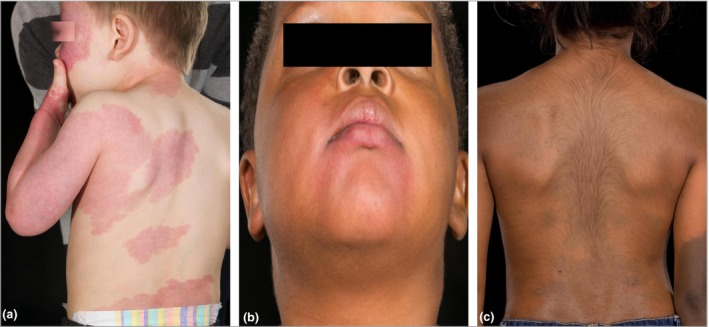
Cutaneous phenotypic spectrum of mosaic G‐protein disorders. Patients with identical mosaic mutations in *GNAQ* (codon 183), presenting phenotypically with (a) Sturge–Weber syndrome, (b) phakomatosis pigmentovascularis and (c) extensive dermal melanocytosis.

A review of the clinical records was used to complete missing data where possible. To ensure that the common occurrence of self‐resolving Mongolian blue spots did not bias our data collection, dermal melanocytosis was considered to be relevant only where it was ‘atypical’. On the basis of observational studies of normal Mongolian blue spots^4^, ^5^ we have defined this as fulfilling any two of the following criteria: (i) involvement of sites other than only the lumbosacral area, (ii) persistence beyond the first 2 years of life, (iii) areas > 10 cm in diameter at birth and (iv) some areas of accentuated deep pigmentation with clearly defined borders. Examples of the clinical phenotypes for PPV and EDM are shown in Figure 1.

**Figure 2:**
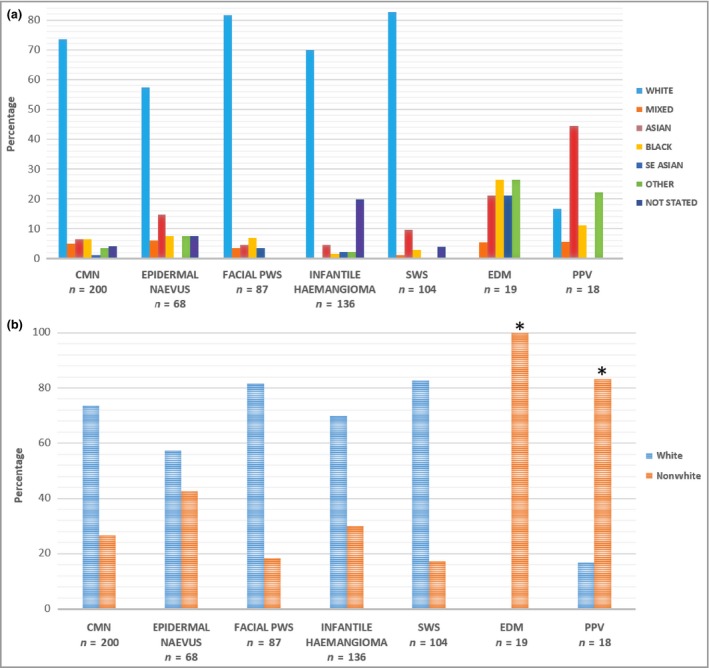
(a) Ethnicity of patients with different birthmarks attending Great Ormond Street Hospital paediatric dermatology department. CMN, congenital melanocytic naevus; PWS, isolated port‐wine stain; SWS, Sturge–Weber syndrome; EDM, extensive dermal melanocytosis; PPV, phakomatosis pigmentovascularis. Patients who self‐reported in the ‘other’ ethnicity cohort were predominantly of Middle Eastern descent. This is not currently offered as an ethnic category in our hospital's admission documentation, which uses standardized National Health Service ethnic category codes derived from the classification used by the Office of National Statistics for the 2001 census. (b) Ethnicity profile of different birthmarks comparing white vs. nonwhite ethnicity. *P < 0·001 using Fisher's exact test.

The percentages of different ethnicities in our whole referral cohort revealed a substantial preponderance of white ethnicity in all birthmark groups, as would be expected for our geographical referral population, except for PPV and EDM (Fig. 2a). In these birthmark groupings there were significantly fewer white patients; there were none in the EDM cohort and only three out of 18 in the PPV cohort. Although the numbers in these groups are relatively low, they are comparable with the numbers of nonwhite patients in all other birthmark groupings. Furthermore, statistical comparison of PPV or EDM and all other birthmark groupings revealed a significant difference in white vs. nonwhite ethnicity (*P* < 0·001, Fig. 2b). When only the port‐wine stain, SWS, PPV or EDM birthmarks were analysed (restricting the analysis to these, as we know they can be caused by *GNAQ* or *GNA11* mosaicism) the same pattern is in evidence; logistic regression modelling of having a pigmentary component to the cutaneous phenotype produced an odds ratio for white ethnicity of 0·017 (95% confidence interval 0·005–0·060, *P* < 0·001). There was no effect of sex.

The mechanism of this association between cutaneous phenotype within this diagnostic spectrum and ethnicity is not yet clear. However, we hypothesize that germline ethnicity‐associated variants in pigment genes could be involved, perhaps including the melanocortin‐1 receptor gene *MC1R*, which encodes a G‐protein‐coupled receptor. Variants in *MC1R* are already known to modify the phenotype of congenital pigmentary disorders such as oculocutaneous albinism and congenital melanocytic naevi.^6^, ^7^ Furthermore, although the canonical signalling pathway from MC1R is via cyclic AMP, there is some evidence that signalling via calcium release can occur,^8^, ^9^ which could support this hypothesis of coupling of MC1R to Gα_q_ and Gα_11_.

In conclusion, postzygotic mosaicism for *GNA11* and *GNAQ* mutations causes an overlapping phenotypic spectrum of vascular and melanocytic birthmarks, with associated ophthalmological, neurological, overgrowth and malignant complications. Ethnicity appears to be associated with congenital phenotypic variation in the cutaneous component of this spectrum of mosaic disease.
